# Classroom Behavior Detection Method Based on PLA-YOLO11n

**DOI:** 10.3390/s25175386

**Published:** 2025-09-01

**Authors:** Hongshuo Zhang, Guohui Zhou, Wei He, Hanlin Deng

**Affiliations:** School of Computer Science and Information Engineering, Harbin Normal University, Harbin 150025, China

**Keywords:** classroom behavior detection, YOLOv11, PConv, LSKA, AIFI

## Abstract

Accurate detection of student behavior in the classroom helps analyze students’ learning states and contributes to improving teaching effectiveness. We propose the PLA-YOLO11n classroom behavior detection model. We design a novel C3K2_PConv module that integrates partial convolution with modules from the YOLO11 network and apply it to the backbone and neck feature fusion layers. To enhance small-target feature representation, we incorporate a large-kernel self-attention (LSKA) mechanism and replace the SPPF at the end of the backbone with the attention feature integration module (AIFI). We also add a high-resolution detection head. Experimental results on the SCB2 dataset demonstrate that the improved model outperforms the original YOLO11, achieving an increase of 3.8% in mean average precision (mAP@0.5).

## 1. Introduction

With the advancement of educational psychology and cognitive science, the theory of Self-Regulated Learning (SRL) has become a critical framework for understanding students’ learning processes and the motivational mechanisms underlying their behaviors [1]. SRL emphasizes learners’ active regulation of their learning activities across four stages—goal setting, strategy implementation, process monitoring, and outcome reflection—thereby fostering continuous improvement in learning outcomes [2]. In this process, timely and accurate acquisition of learning behavior data can provide immediate feedback to learners and assist teachers or intelligent educational systems in dynamically adjusting instructional strategies. However, traditional classroom behavior monitoring relies primarily on teachers’ visual observation and post-class video analysis. Both approaches are susceptible to subjective bias and attentional limitations and cannot provide continuous, quantitative tracking of subtle behaviors across the entire class, resulting in delayed assessments that fail to meet the demands of personalized instruction [3].

To overcome the limitations of traditional approaches, researchers have increasingly turned to Deep Learning [4] and Computer Vision [5] technologies. Compared with traditional image processing methods that depend on manually designed features, deep learning approaches can automatically learn multi-level feature representations from large-scale data, significantly enhancing robustness in realistic classroom scenarios involving small targets and occlusions [6]. Its core advantages include (1) an end-to-end framework that eliminates the need for complex manual feature engineering; (2) convolutional networks that progressively capture visual information ranging from local motion to global spatial layout through multi-scale receptive fields; and (3) the integration of attention mechanisms to dynamically focus on key regions (e.g., hand-raising, head-down, interactive gestures) while suppressing background noise, thereby enhancing the accuracy and stability of action detection and classification [7,8,9].

Currently, research methods for classroom student behavior recognition can be broadly categorized into three types: video action recognition approaches [10], human pose estimation approaches [11], and end-to-end detection frameworks based on deep convolutional neural networks (DCNNs) [12]. Video action recognition methods typically employ spatiotemporal convolutions (3D CNNs) [13] or two-stream networks [14] to classify behaviors holistically. For example, Tan et al. [15] investigated several typical classroom actions such as standing, hand-raising, and writing. However, such methods often depend on large volumes of annotated video data and exhibit inaccuracies in determining action boundaries and durations [16]. Pose estimation-based methods extract skeletal joint sequences via human keypoint detection algorithms and use graph neural networks or temporal models to classify behaviors. For instance, in 2016, Cao et al. [17] proposed a real-time multi-person 2D pose estimation method that employed PAFs to learn associations between body parts, enabling efficient and accurate human pose estimation. In educational scenarios, however, factors such as dense limb occlusions and subtle changes in small-scale targets often result in unstable keypoint detection, thereby affecting downstream behavior classification performance [18]. In contrast, single-stage and two-stage detection methods based on DCNNs can directly produce behavior detection results at the image or video-frame level while simultaneously performing detection and classification, making them the mainstream approaches in current research and industrial applications [19].

From a more detailed perspective, current object detection approaches can be broadly divided into two categories: two-stage methods and single-stage methods [20]. Among these, the two-stage detection method is represented by Faster R-CNN [21], whose workflow typically involves two main steps: first, generating candidate regions, and then performing feature extraction and classification regression on these regions. The R-CNN framework proposed by Girshick et al. [22] in 2014 pioneered the use of convolutional neural networks for object detection tasks, laying the foundation for two-stage detection methods. Furthermore, Faster R-CNN, introduced by Ren et al. [23] in 2015, integrated the RPN to achieve an end-to-end detection pipeline, significantly improving both speed and accuracy. However, since such methods introduce large computational overhead in candidate box generation and multi-stage processing, their actual deployment and application are limited in scenarios with high real-time requirements, such as classroom behavior recognition [24].

By contrast, single-stage detection algorithms, including SSD [25], RetinaNet [26], and the YOLO (You Only Look Once) family [27], adopt a more compact architecture. Rather than generating candidate regions as an intermediate step, these models directly perform parallel bounding-box regression and category probability prediction on the feature maps. This unified end-to-end design substantially boosts inference speed, making single-stage algorithms the primary choice for real-time classroom behavior monitoring [28]. In particular, the YOLO framework has undergone multiple iterations since its initial release, achieving ever-higher detection accuracy while maintaining extremely fast speeds. Its ability to perform rapid inference on standard GPU hardware has established a solid foundation for large-scale deployment in smart classroom systems [29].

To further enhance the performance of the one-stage detection architecture in complex classroom behavior recognition tasks, researchers continue to carry out structural optimization and strategy improvements in the YOLO series models. For example, Zhou et al. [30] proposed CSSA-YOLO, a cross-scale spatiotemporal attention network that integrates C2f (Cross Stage Partial Network Fusion) modules, Shuffle Attention, and WIoU (Weighted IoU) loss into YOLOv8, significantly improving the accuracy and real-time performance of fine-grained classroom behavior recognition. Jia et al. [31] developed a hybrid detection framework based on YOLOv5-CA (Coordinate Attention). The model effectively enhanced the feature expression ability of the back row students’ actions through the spatial-channel collaborative attention characteristics of the coordinate attention mechanism. Although single-stage detectors excel in both speed and accuracy, they still struggle to reliably distinguish diverse behaviors in complex classroom environments and often underperform in recognizing small targets under occlusion or low-light conditions [32]. Future research should focus on enhancing the scene adaptability and stability of the model, improving the occlusion processing strategy, and optimizing the small target detection performance.

At present, detection performance in complex classroom environments still faces two major challenges. First, frequent occlusions and overlaps in classroom scenes severely hinder behavior recognition. The close spatial proximity of students, combined with obstructions from desks, chairs, and books, often renders parts of the behavior subject invisible. This makes it difficult for detection models to accurately identify individual actions, especially when multiple students raise their hands or stand simultaneously, leading to misclassification or missed detections. Second, many critical classroom behaviors manifest as subtle, small-scale actions, such as lowering the head, writing, or checking a phone. Such small targets are not only tiny and short-lived but also often share similar colors with the background or clothing, further complicating feature extraction and classification. These issues limit the generalization and accuracy of existing detection models in complex teaching environments. Thus, improving the robustness of behavior recognition under occlusion and enhancing sensitivity to fine-grained actions have become key technical challenges for effective classroom behavior analysis.

To address the aforementioned challenges, we propose the PLA-YOLO11n algorithm for classroom behavior detection, which achieves high-accuracy recognition through multi-dimensional algorithmic optimization and provides quantitative support for teacher evaluation. The model is built upon the YOLO11 architecture and integrates multi-level feature enhancement to effectively mitigate common issues in complex classroom environments, such as small-target feature loss and dense occlusions. We adopt YOLO11n as the baseline for evaluation, as it offers a favorable trade-off between detection accuracy and computational cost on the SCB2 (SCB-Dataset 2) dataset. As the latest iteration of the YOLO family, YOLO11 introduces additional architectural refinements over previous versions, delivering improved detection accuracy for classroom behavior tasks while further reducing computational overhead. Experimental results demonstrate a 3.8% improvement in mAP@0.5 compared with the baseline model, validating the practical value of the proposed method for assessing teaching quality and monitoring students’ learning states in real-world classroom scenarios. The main contributions of this work are as follows:

- The partial convolution (PConv) is deeply integrated into the C3K2 (CSP Bottleneck with 3 × 3 and 5 × 5 Convolutions) module to build a dynamic sparse connection structure in the channel dimension. This module improves the performance and robustness of the detection model.
- The Large Separable Kernel Attention (LSKA) module is embedded into the neck network, and the correlation between local behavior and global semantics is strengthened through long-range dependency modeling, which effectively improves the model’s detection capabilities in occlusion problems and fine-grained target detection.
- The SPPF module is replaced by the Attention-based Intra-scale Feature Interaction (AIFI) to further improve the model’s ability to perceive objects at different scales.
- The newly added P2 detection head establishes a shallow feature pass-through mechanism, which significantly improves the small target detection performance of the model.
- We tested the PLA-YOLO11n model on the SCB2 dataset. Compared with the baseline model, the proposed method improved the mAP@0.5 by 3.8% in dense scenes, outperforming the existing mainstream algorithms.

The remainder of this paper is organized as follows: Section 2 provides an overview of the relevant techniques, Section 3 describes our proposed method, Section 4 presents the experiments and discusses the results, and Section 5 concludes the work and outlines future research directions.

## 2. Related Work

### 2.1. YOLO11 Framework

YOLO11 [33] was officially released on 27 September 2024, by Glenn Jocher and Jing Qiu from the Ultralytics team. As the latest generation of real-time object detection models in the YOLO family, it continues the lightweight and efficient design philosophy while achieving significant improvements in architectural optimization and inference efficiency. YOLOv11 maintains detection accuracy while further reducing computational resource consumption, demonstrating strong adaptability in complex classroom environments and making it particularly suitable for applications with stringent real-time and accuracy requirements.

**Figure 1:**
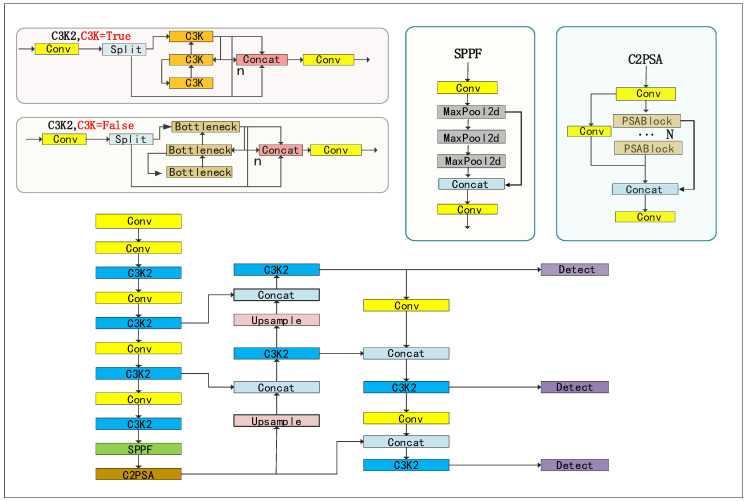
YOLOv11 network architecture.

As shown in Figure 1, the overall architecture of YOLOv11 consists of three core components: the backbone, the neck, and the detection head. Each module incorporates the latest lightweight designs and enhanced feature representation strategies, aiming to improve the model’s ability to detect fine-grained targets while maintaining high inference speed. For clarity, the figure further illustrates four key functional modules: the top-left section presents two implementations of C3K2, which strengthen local feature representation and reduce computational redundancy through stacked convolutions and cross-layer connections; the top-center section depicts the SPPF module, designed to efficiently aggregate multi-scale features and thus enhance inference efficiency; on the right, the C2PSA (Cross Stage Partial Pyramid Squeeze Attention) module employs a selective attention mechanism to suppress background noise and highlight critical target regions. Finally, the bottom illustrates the overall connection flow of the Backbone–Neck–Head, providing an intuitive view of how these functional modules are embedded within the network.

In the backbone, YOLOv11 introduces the C3K2 module, which leverages stacked repetitive convolutions and cross-layer connections to enhance local feature representation while reducing parameter redundancy and computational load. Additionally, the C2PSA module employs selective attention to suppress background noise and guide the network to focus on key target regions, significantly improving discriminative ability in complex backgrounds. The neck utilizes a bidirectional Path Aggregation Feature Pyramid Network (PAFPN), combining top-down feature fusion with bottom-up path augmentation to effectively balance detail and semantic information across multiple scales. The detection head adopts decoupled prediction to separate classification and regression tasks and integrates depthwise separable convolutions to reduce computational overhead, thereby enhancing both robustness and inference efficiency.

However, when applied to real classroom scenarios, challenges remain in handling densely populated video frames, severe inter-person occlusions, and the detection of small targets. To address these issues, we introduce a series of enhancements to YOLOv11, including an improved C3K2 structure, the Large Separable Kernel Attention (LSKA) mechanism, replacing SPPF (Spatial Pyramid Pooling—Fast) with the AIFI (Attention-Based Intra-Scale Feature Interaction), and adding a P2 (used for small object detection) detection head. These modifications significantly improve detection accuracy and robustness for small targets and occluded behaviors in complex classroom environments.

### 2.2. Partial Convolution (PConv)

In convolutional neural networks, conventional full-channel convolutions introduce substantial redundant computation when processing high-dimensional feature maps, particularly when information across channels is highly repetitive. This leads not only to inefficient use of computational resources but also to degraded inference efficiency. To alleviate this problem, the partial convolution mechanism was proposed. The core idea is to select only part of the channels to participate in the spatial convolution calculation in the convolution operation, thereby reducing the computational complexity and memory access.

**Figure 2:**
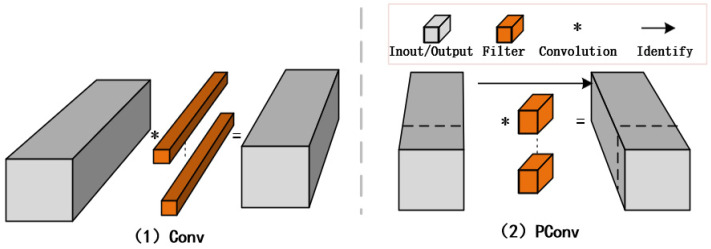
Design principle of PConv.

The core mechanism of PConv lies in a sparse computation strategy. For each input feature map, a binary mask is maintained: only channels with a mask value of 1 undergo *k* × *k* convolution, while channels with a mask value of 0 are preserved through an identity mapping. The convolution outputs are normalized according to the number of participating channels to ensure consistent output magnitudes. As shown in Figure 2, this structure effectively implements “mask-guided partial channel convolution”, enhancing both adaptability to occluded regions and numerical stability.

In addition, to enhance network representational capacity, PConv is typically combined with 1 × 1 pointwise convolution, which performs subsequent full-channel fusion to compensate for potential information loss from sparse convolution. This two-stage design balances computational efficiency with semantic completeness and is highly deployable and hardware-friendly.

Specifically, assuming that the input feature map size is *h* × *w*, the number of channels is *c*, the number of channels involved in the convolution is *c_p_*, and the convolution kernel size is *k*, the partial convolution ratio *r* is defined as:

(1) $$r=\frac{c_{p}}{c}$$

The corresponding computational cost (FLOPs) and memory access cost (MAC) can then be expressed as:

(2) $$FLOPs_{PConv}=h\cdot w\cdot k^{2}\cdot c_{p}$$

(3) $$MAC\approx h\cdot w\cdot c_{p}+k^{2}\cdot c_{p}\cdot c$$

In a typical configuration, if *r* = 1/4, the computational cost and memory access can be reduced to one-quarter or less compared to standard convolution, effectively alleviating resource constraints during inference. Furthermore, PConv often employs a subsequent 1 × 1 pointwise convolution to achieve cross-channel fusion, enabling preserved feature information to propagate across all channels and thereby balancing computational efficiency with representational completeness.

PConv’s masking mechanism can further enhance feature selectivity. In some implementations, PConv introduces a binary mask on the input feature map, performing convolution operations only on channels or pixel locations where the mask value is 1, while maintaining an identity mapping for locations where the mask is 0. By updating the mask state layer by layer, it achieves gradual “completion” of missing or occluded regions and semantic guidance.

In object detection, particularly for classroom behavior recognition involving severe occlusions or densely packed small targets, PConv serves as a low-redundancy, high-efficiency alternative to standard convolutions. It effectively focuses on information-rich channels, suppresses background and redundant features, and significantly improves both bounding-box localization accuracy and classification performance.

### 2.3. Attention-Based Intra-Scale Feature Interaction

Zhao et al. [34] proposed the Attention-based Intra-scale Feature Interaction (AIFI) mechanism, which is based on the Transformer encoder structure. Through 2D positional encoding and a multi-head self-attention mechanism, AIFI establishes deep semantic relationships and global context modeling within the same scale, thereby enhancing the model’s ability to represent fine-grained behaviors and multi-scale targets in complex scenarios.

Specifically, assuming that the high-level feature map output by the backbone network is $S\in R^{C\times H\times W}$, AIFI first expands it in the row direction and converts it into a two-dimensional serialized feature matrix $X\in R^{L\times C}$ (where *L* = *H* × *W*), and injects two-dimensional spatial position information through the position encoding mechanism to enhance the spatial recognizability of the feature. Subsequently, a linear transformation is used to generate the query, key, and value vectors required by the attention mechanism:

(4) $$Q=W_{Q}X,\;\;K=W_{K}X,\;\;V=W_{V}X$$

Among them, $W_{Q},W_{K},W_{V}\in R^{C\times d}$ are learnable parameter matrices, and *d* represents the attention dimension. AIFI then uses the multi-head self-attention mechanism to calculate the scaled dot-product attention:

(5) $$Attention(Q,K,V)=softmax(\frac{QK^{T}}{\sqrt{d}})V$$

This mechanism allows the model to capture interactions between distant elements within the same-scale features, effectively enhancing interaction modeling between regions such as student posture, gestures, and facial behaviors. The outputs of all attention heads are then concatenated and reshaped into a spatial feature map with the same dimensions as the input. These are passed through a multi-layer perceptron (MLP) and layer normalization (LayerNorm) modules for feature enhancement, ultimately being concatenated with global pooling features to form the semantically enhanced output.

Unlike conventional feature fusion modules, AIFI’s structure emphasizes long-range semantic modeling and region-dependent interactions within a single scale, avoiding the redundancy and computational overhead caused by multi-scale stacking. This mechanism is particularly well-suited for dynamically modeling subtle movements in student behavior, offering stronger spatial adaptability and representational robustness. Its applicability in real-world teaching scenarios provides the theoretical foundation for the network improvements proposed in subsequent sections.

### 2.4. Large Separable Kernel Attention

In complex visual tasks, attention mechanisms assign dynamic weights to input features. This allows the model to emphasize key information while suppressing redundant noise. As a result, detection and recognition performance is significantly improved. The classic Large Kernel Attention (LKA) introduces *k* × *k* convolution kernels and dilated depthwise separable convolutions. These expand the receptive field and capture long-range spatial dependencies. Given an input feature map $F\in R^{C\times H\times W}$, the basic operation of LKA is shown in Equation (6):

(6) $$Z=W_{k\times k}*F,\;A=\sigma (W_{1\times 1}*Z),\;\tilde{F}=A⊙F$$

Here, * denotes spatial convolution, ⊙ represents element-wise multiplication, σ(·) is the Sigmoid activation function, and *W_k_*_×*k*_ and *W*_1×1_ represent the large kernel convolution and pointwise convolution weight matrices, respectively. LKA effectively expands the receptive field and enhances long-range information modeling, but its computational cost and parameter size grow quadratically with the kernel size *k*^2^, limiting real-time performance for high-resolution inputs.

To address this, the Large Separable Kernel Attention (LSKA) module performs structural optimization on LKA. Its core idea is to retain the ability to model large receptive fields while decomposing the 2D large kernel convolution into two 1D convolution operations. First, a horizontal separable convolution is applied to the input feature *F*, as shown in Equation (7):

(7) $$Z^{\prime }=DWConv_{1\times k}(F)$$

This process captures long-range dependencies in the horizontal direction of the feature map. Subsequently, a vertical separable convolution is applied to further model the vertical semantics, as shown in Equation (8):

(8) $$Z=DWConv_{k\times 1}(Z^{\prime })$$

To further improve contextual coverage, the LSKA module incorporates dilated convolutions into the above convolution operations, expanding the receptive field without noticeably increasing the number of parameters. After the bidirectional convolution, the feature map *Z* is passed through a 1 × 1 pointwise convolution and activated to produce a spatial attention map A. This map is then element-wise multiplied with the input feature F to recalibrate it, yielding the final output, as shown in Equation (9).

(9) $$A=\sigma (W_{1\times 1}(Z)),\;\tilde{F}=A⊙F$$

By replacing the 2D large kernel convolution with a series of 1D convolution operations, LSKA reduces the original computational complexity from O(*k*^2^) to O(2*k*), significantly alleviating the network’s computational burden. At the same time, the module retains efficient modeling of long-range spatial dependencies and possesses strong channel and spatial adaptivity.

In summary, LSKA provides the theoretical and structural foundation for its efficient integration into the YOLO11 detection framework. While maintaining computational efficiency, it significantly enhances the model’s spatial awareness and effectively improves its detection performance for occlusion and small target behaviors.

## 3. Methodologies

### 3.1. Overall Framework

**Figure 3:**
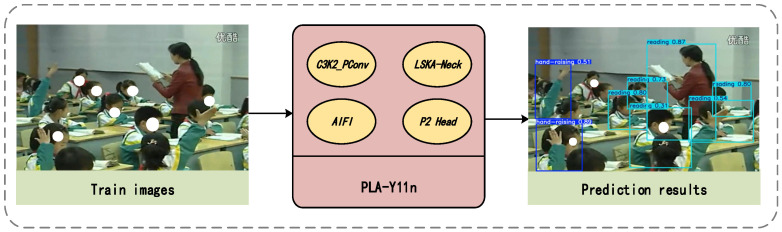
Overall framework for classroom behavior detection.

This section provides a detailed overview of the overall framework structure, as shown in Figure 3. This study proposes the PLA-YOLO11n model for classroom behavior detection and analysis. The original training images, processed through the optimized network, enable accurate recognition of students’ classroom behaviors. In Section 3.2.1, we provide a detailed description of the design of the C3K2_PConv module. Section 3.2.2 explains the introduction of the LSKA module. Section 3.2.3 outlines the optimization and improvements made to the feature pyramid structure. Section 3.2.4 introduces a new high-resolution detection head. The entire framework achieves end-to-end classroom behavior analysis functionality through a modular design.

### 3.2. Improved PLA-YOLO11n Model

**Figure 4:**
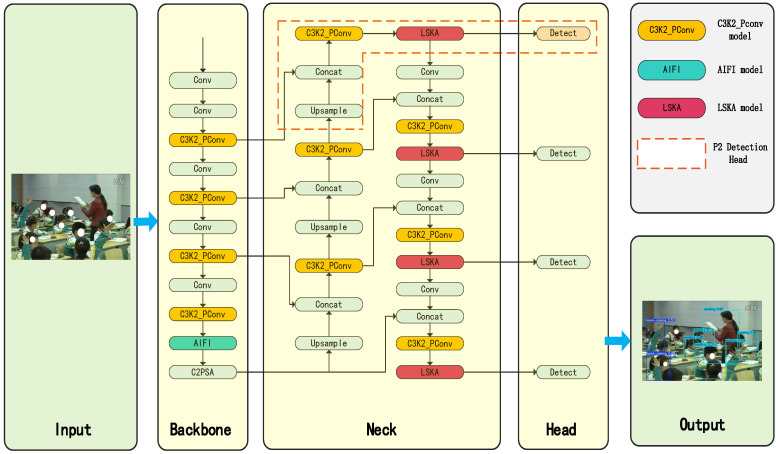
Improved YOLO11 network framework.

To improve the accuracy of classroom student behavior detection, we propose an improved YOLOv11-based detection model, PLA-YOLO11n, as shown in Figure 4. This model innovatively incorporates the Partial Convolution (PConv) mechanism to construct a lightweight feature extraction C3K2_PConv module, enabling efficient feature selection and compression within the backbone network. In the feature fusion stage, we designed the Large Separable Kernel Attention (LSKA) mechanism with a large receptive field to enhance spatial relationship modeling, while optimizing multi-level feature fusion with the Attention-based Intra-scale Feature Interaction (AIFI) module. The detection output extends the feature pyramid structure, with the addition of a shallow detection path that significantly improves the recognition of fine-grained targets. The improved network architecture provides a complete end-to-end processing pipeline from image input to behavior recognition, achieving significant improvements in detection accuracy while maintaining real-time performance. Experimental validation shows that this model effectively addresses challenges such as occlusion and small target detection in classroom scenarios.

#### 3.2.1. C3K2_PConv Module

To further improve the inference efficiency and feature sensitivity of the YOLOv11 network in classroom behavior recognition tasks, this paper proposes an improvement to the C3K2 module in its backbone network by introducing the Partial Convolution (PConv) mechanism described in Section 2, thereby constructing a lightweight feature extraction structure, C3K2_PConv.

The design motivation for this structure lies in the fact that, in the traditional C3K2 module, all channels participate in spatial convolution operations, leading to high computational costs and redundancy in some channels. The C3K2_PConv module introduces partial convolution in the bottleneck structure, activating only about a quarter of the channels for convolution calculation, while the remaining channels are retained through identity mapping, and then 1 × 1 point convolution is used to complete cross-channel information fusion, thereby taking into account both feature transfer integrity and inference efficiency.

Additionally, since PConv has stronger focusing capabilities on key regions in the spatial dimension, it is more robust in extracting detailed features such as occlusions in the classroom, head postures, and writing actions. Experiments show that after replacing the original module with the C3K2_PConv module, the model improves overall inference speed while maintaining accuracy, and the number of parameters decreases, effectively enhancing the model’s deployment value in real-world scenarios.

**Figure 5:**
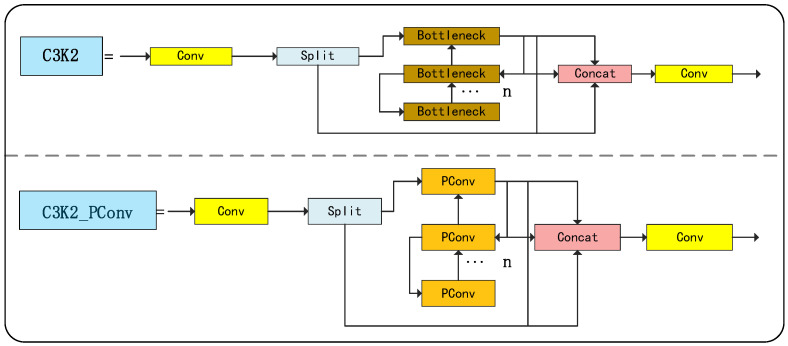
Comparison of C3K2_PConv and the original module structure.

As shown in Figure 5, the C3K2_PConv retains the original module’s backbone path design while using PConv to replace conventional convolutions in the Bottleneck branch, forming a novel feature extraction unit that combines sparse channel computation with full-channel representation, further optimizing network performance.

#### 3.2.2. Replacing SPPF with AIFI

In the original YOLOv11, deep semantic fusion relies on the Spatial Pyramid Pooling Fast (SPPF) module, which improves perception of targets at different scales through multi-scale max pooling. However, its static stacking method fails to model long-range dependencies within the feature map. In classroom scenarios, occlusions, irregular layouts, lighting changes, and varying viewpoints often cause semantic conflicts or loss of details in features at different scales for the same behavior, particularly leading to missed detections for fine actions such as writing, hand-raising, or when local occlusions occur. To address this, we replace SPPF with the Attention-based Intra-scale Feature Interaction (AIFI) module, which performs semantic modeling within a single scale on deep features (e.g., S5) using 2D positional encoding and multi-head self-attention. This dynamic approach captures spatial context relationships and significantly enhances the model’s ability to structurally recognize fine-grained behaviors in complex scenes.

**Figure 6:**
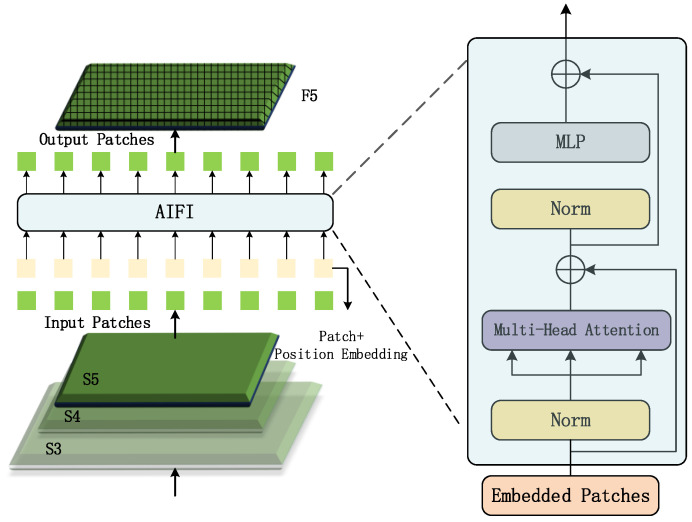
AIFI structure diagram.

As shown in Figure 6, AIFI first serializes high-level features and embeds 2D positional information, followed by learning spatial interactions between positions through the attention mechanism. Unlike the stacking approach of SPPF, which is based on fixed-scale pooling kernels, AIFI adapts to focus on key behavior regions of students based on feature distribution, suppressing redundant background noise and improving detection discriminability. Moreover, the output structure of AIFI maintains consistency with the original feature map, allowing for seamless integration with the backbone network, and imposes almost no additional computational burden, offering good deployment compatibility.

From an overall structural perspective, the AIFI offers three key advantages: first, it builds long-range context dependencies within the same scale, overcoming the limitations of receptive fields in traditional convolutions and pooling operations; second, it possesses dynamic semantic focusing capabilities, allowing the model to actively learn global response patterns between behavior regions; third, its structure is simple, with limited parameter increments, enabling it to directly replace the original SPPF module and be easily integrated into existing detection frameworks. Considering the specific needs of teaching scenarios, AIFI exhibits stronger robustness for small targets, action occlusion, and background complexity.

Experimental results demonstrate that, while maintaining the original network architecture and inference speed, the YOLO11 model with AIFI significantly improves accuracy in complex classroom behavior detection, particularly in dense occlusion or frequent fine-movement scenarios, showing more stable performance. As shown, the introduction of AIFI not only enhances the feature expression capability of the backbone network but also provides an effective structural optimization path for building high-performance object detection systems tailored to real-world teaching environments.

#### 3.2.3. Neck Network with LSKA

To enhance the spatial modeling capability of YOLO11 in complex classroom scenarios, we introduce the Large Separable Kernel Attention (LSKA) module into the original network structure and integrate it between the Neck and Head components. This module is used to perform spatial recalibration on the fused multi-scale feature maps, thereby emphasizing regions related to student behaviors while suppressing background interference.

**Figure 7:**
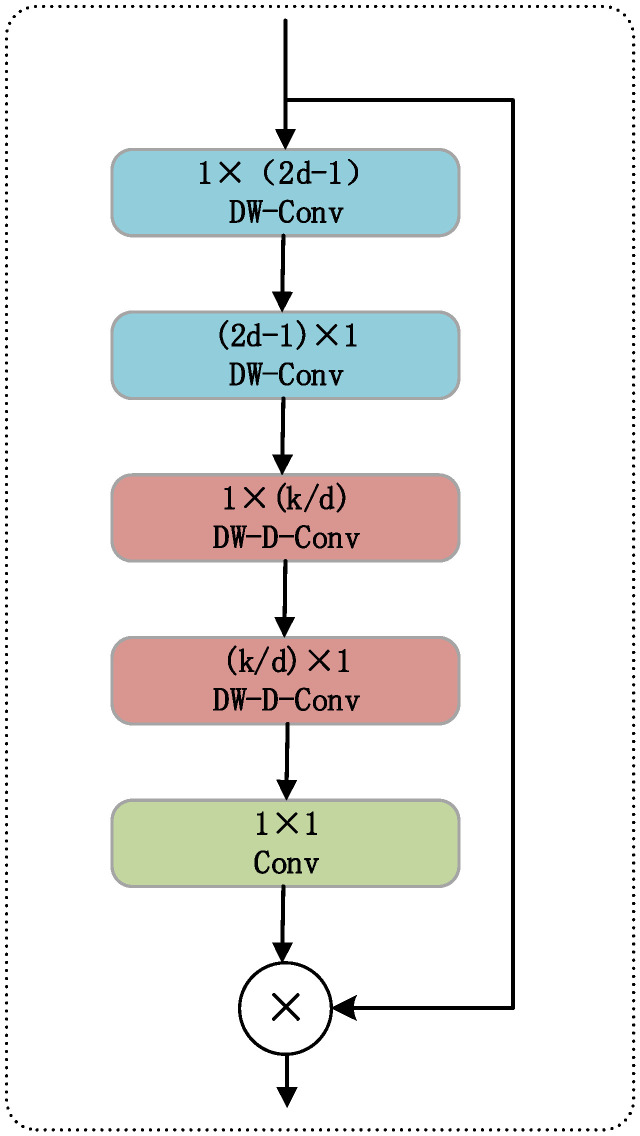
LSKA structure diagram.

As shown in Figure 7, LSKA receives the output feature map $F\in R^{C\times H\times W}$ from Neck, and successively passes it through 1 × *k* and *k* × 1 separable depth convolutions to model the spatial dependencies in the horizontal and vertical directions. To further expand the receptive field, dilated convolution can be introduced. Afterwards, a 1 × 1 pointwise convolution and Sigmoid activation are used to generate the spatial attention map, which is then element-wise multiplied with the original feature map to obtain the recalibrated feature $\tilde{F}$, serving as the input to the detection head.

Compared to traditional methods, the LSKA module effectively enhances the network’s ability to perceive behavior-related areas without significantly increasing parameters or computational cost. It demonstrates stronger discriminability and robustness, particularly in scenarios with occlusion, complex backgrounds, or small action amplitudes. Moreover, the module is structurally simple, with a clear insertion point, and does not interfere with other parts of the backbone network or detection head, making it convenient for engineering deployment and model optimization.

In summary, by incorporating the LSKA module into the detection path of YOLO11, the model gains enhanced spatial feature expression capabilities, which help improve the accuracy and stability of classroom behavior detection.

#### 3.2.4. Addition of P2 Detection Head

In smart classroom scenarios, behaviors such as students raising hands, writing, or nodding often occupy very small pixel areas in images, making it difficult for the standard YOLO11 structure to stably detect these small targets. YOLO11 by default sets up P3 (80 × 80), P4 (40 × 40), and P5 (20 × 20) detection heads on three feature maps from the Backbone, where the deeper P5 captures strong semantic information suitable for large-scale targets, and the shallower P3 retains more spatial details, but its resolution is still insufficient to accurately locate smaller gestures and writing actions in the classroom.

To enhance sensitivity to small behaviors, we introduce an additional P2 detection head with a resolution of 160 × 160, in addition to the original three detection heads. The implementation steps are as follows: First, shallow features are directly extracted from the second-stage feature map output from the Neck (downsampled by a factor of 4, with a size of 160 × 160), ensuring the retention of complete spatial details. Then, the feature map undergoes convolutional fusion to unify the channel count and feature representation format. The fused P2 features are then passed into the newly added detection head branch, where the same class and bounding box predictions as P3–P5 are executed. This design enables the network to simultaneously account for shallow high-resolution details and deep strong semantic representations during the learning process.

The newly added P2 detection head shares the same set of output head structures as the original detection head, but because it predicts shallow high-resolution inputs, it can more accurately capture the position and contours of small targets of classroom behavior. By incorporating more supervision from small target samples during training, the P2 branch effectively compensates for the accuracy shortcomings of P3–P5 in small target detection, thereby enhancing the model’s ability to recognize occlusions and fine-grained behaviors. Experimental results show that, while slightly reducing overall inference speed, this improvement significantly enhances the recall rate and localization accuracy of classroom behavior detection, providing more reliable technical support for smart classroom behavior analysis.

## 4. Experiments

### 4.1. Datasets and Evaluation Metrics

#### 4.1.1. Dataset

**Figure 8:**
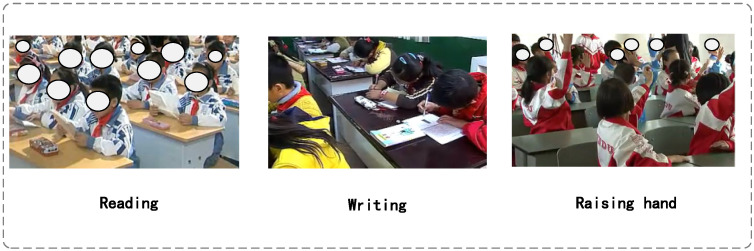
Key characteristics of the SCB2 dataset.

To verify the effectiveness of the proposed YOLO11-based classroom behavior detection method in practical application scenarios, we conducted experimental evaluations using the publicly available SCB2 dataset [35]. This dataset was specifically constructed for behavior recognition tasks in classroom surveillance settings, offering strong representativeness and diversity. It contains approximately 4200 images with 18,499 behavior annotations, covering three typical classroom behaviors—reading, writing, and raising hands—as illustrated in Figure 8. These three behaviors not only capture key characteristics of classroom interaction but also present common detection challenges such as small object scale and frequent occlusions, thereby imposing higher demands on the model’s localization and spatial discrimination capabilities.

Notably, the dataset contains a substantial number of small-scale targets, particularly in scenarios such as students raising their hands or reading with their heads down, where hand and facial regions occupy only a tiny portion of the image and are often obscured by other students, desks, chairs, or lighting conditions. Therefore, the SCB2 dataset plays a critical role in validating the performance of the newly introduced detection head structure and spatial attention mechanism, providing a rigorous benchmark for assessing the model’s robustness in handling small objects and occluded behaviors.

**Figure 9:**
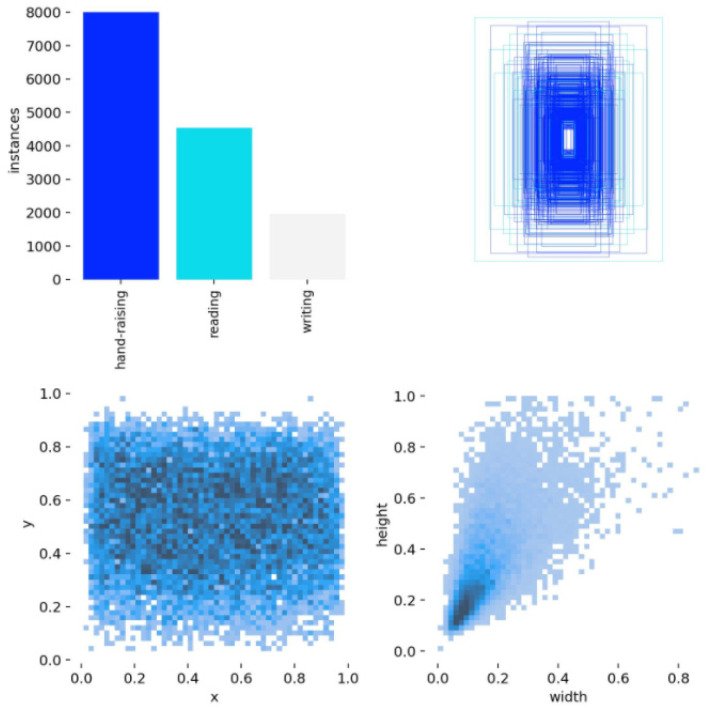
Target distribution of the SCB2 dataset.

In the experiments, we adopted the official training and validation split of the SCB2 dataset, which partitions the data into training and validation sets at a 4:1 ratio without modification, thereby ensuring the fairness and objectivity of the results. The dataset and its official split are publicly available at: [https://github.com/Whiffe/SCB-dataset (accessed on 26 August 2025)]. The distribution of the dataset’s target categories is shown in Figure 9.

Figure 9 presents the statistical analysis of the SCB2 dataset. The bar chart on the left illustrates the distribution of the three behavior categories—”raising hands”, “reading”, and “writing”—with approximately 8000, 4000, and 2000 samples, respectively, highlighting the imbalance in sample sizes across categories. The scatter plots in the middle and on the right further depict the distribution of bounding-box parameters, with most data points concentrated in lower-value regions. This indicates that the dataset contains a large number of small-scale objects, providing a solid foundation for investigating methods for detecting small objects.

Overall, the SCB2 dataset, with its realistic classroom perspectives and diverse small-scale behavior samples, provides a reliable data foundation and experimental basis for classroom behavior detection tasks, and is particularly well suited for research on methods targeting occluded and small-scale actions.

#### 4.1.2. Evaluation Metrics

To comprehensively and objectively evaluate the performance of the proposed classroom behavior detection model, we adopted mean Average Precision (mAP), a widely used metric in object detection tasks, as the primary evaluation criterion. In combination with Precision and Recall, we conducted an integrated analysis of the model’s detection performance across different behavior categories.

In behavior detection tasks, true positives (TP) refer to bounding boxes correctly identified as belonging to the target class; false positives (FP) indicate instances where the model incorrectly predicts non-target classes as targets; false negatives (FN) represent instances where the model fails to detect existing target-class samples.

Precision measures the proportion of correctly predicted positive samples among all predicted positives, and is defined as:

(10) $$Preci\sin=\frac{TP}{TP+FP}$$

Recall evaluates the proportion of actual positive samples correctly detected by the model, and is defined as:

(11) $$Recall=\frac{TP}{TP+FN}$$

By plotting the Precision–Recall (P–R) curve at different recall levels and integrating the area under the curve, the Average Precision (AP) for each category can be obtained:

(12) $$AP=\int_{0}^{1}Precision(r)dr$$

To assess the model’s overall detection capability across all behavior categories, the AP values for all classes are averaged to compute the mean Average Precision (mAP):

(13) $$mAP=\frac{1}{C}\overset{C}{\underset{i=1}{\sum }}AP_{i}$$

Here, *C* denotes the total number of behavior categories. A higher mAP value indicates stronger accuracy in multi-class detection. Among the three types of student behaviors involved in this study, namely “reading”, “writing”, and “raising hands”, mAP can be used to comprehensively evaluate the behavior recognition effect of the model in different scenarios, with good adaptability and interpretability.

In terms of efficiency, this study introduced three commonly used indicators. FLOPs (Floating Point Operations) measure the number of floating-point calculations required for a single forward inference, thereby reflecting the model’s computational complexity. Parameters denote the total number of learnable parameters, which indicates the storage overhead and scale of the model. FPS (Frames Per Second) evaluates the inference speed during deployment, representing the number of image frames the model can process per second and thus reflecting its real-time applicability. By jointly considering both accuracy-related and efficiency-related metrics, the evaluation provided a more comprehensive assessment of the model’s suitability and practical value in classroom behavior detection tasks.

### 4.2. Experimental Environment

The configuration environment used in this study is summarized in Table 1, and the hyperparameter settings are detailed in Table 2. To ensure the stability and comparability of the experimental results, all model training procedures were conducted with a fixed random seed of 0.

### 4.3. Experimental Design

To comprehensively evaluate the performance of the proposed PLA-YOLO11n in classroom behavior detection tasks, systematic experiments were conducted on the SCB2 dataset, including ablation studies and comparative experiments with different models, to analyze the contributions of each module (C3K2_PConv, AIFI, LSKA, and the P2 detection head) to the overall performance. To assess the model’s generalization capability across different datasets, additional tests were performed on the SCB3-U and CrowdHuman datasets. All experiments were conducted under the same hardware conditions and consistent training strategies, with a fixed random seed of 0. The values reported in Table 3, Table 4, Table 5 and Table 6 represent the averages of five independent runs, providing more reliable references for performance evaluation.

### 4.4. Experimental Results of PLA-YOLO11n

**Figure 10:**
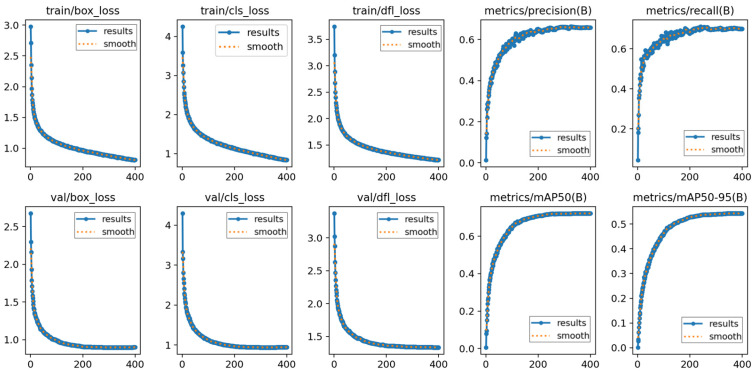
Performance evaluation of the PLA-YOLO11n model.

In this study, the training of the PLA-YOLO11n model was configured for 400 epochs, with an early stopping mechanism triggered once the mean accuracy ceased to show significant improvement. After approximately 320 iterations, the training stabilized, and the improved PLA-YOLO11n model produced its final results on the SCB2 dataset. Figure 10 illustrates multiple performance metrics on both the training and validation sets.

The first three columns in the figure present different loss components during training for the improved PLA-YOLO11n network, including Box Loss, Object Loss, and Classification Loss. Each curve reflects the variation trend of the corresponding loss over the training process, where the X-axis represents training epochs and the Y-axis denotes the loss values. As observed, the overall loss values gradually decrease with training and eventually converge to a stable range. This shows that the proposed PLA-YOLO11n model has good fitting ability and can maintain high stability and accuracy during training.

The last two columns display the Precision–Recall (PR) curves, where the X-axis denotes training epochs and the Y-axis represents precision and recall values. These curves reflect the evaluation of detection performance under different confidence thresholds. As training progresses, the model performance steadily improves, with the curve values approaching 1, indicating a significant increase in detection confidence. The experimental results validate the effectiveness of the PLA-YOLO11n model, demonstrating its stability and efficiency in classroom behavior detection tasks. It consistently improves detection precision and recall in complex scenarios, providing strong support for practical deployment.

**Figure 11:**
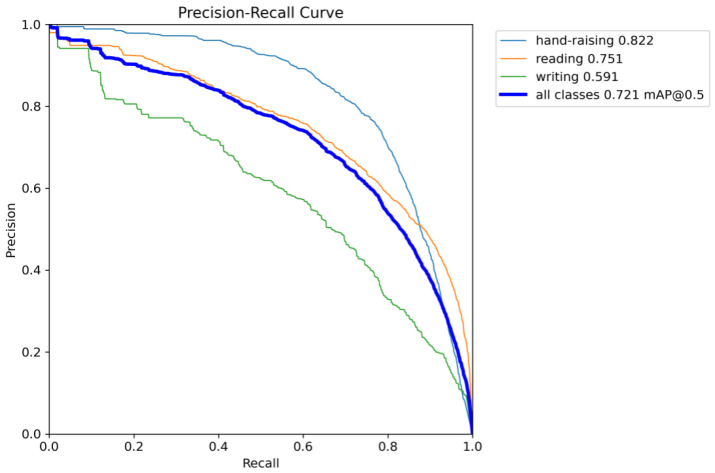
PR curve of the proposed model.

Figure 11 shows the Precision–Recall (PR) curve of the proposed model. In the figure, as recall increases, precision also rises steadily, indicating that the model maintains high detection accuracy across different recall levels. This trend demonstrates that while optimizing recall, the model consistently preserves accurate target recognition, avoiding excessive false positives. The PR curve being close to the upper-right corner indicates that the model achieves a favorable balance between precision and recall, showcasing strong detection capability. The large area under the curve further highlights the model’s superior performance across the entire dataset, effectively covering diverse targets.

Furthermore, the PR curve exhibits a smooth and consistently increasing trend, suggesting a stable relationship between precision and recall across different confidence thresholds. This smoothness implies that the model delivers consistent performance across different target types, avoiding fluctuations or instability in specific categories. Therefore, the proposed PLA-YOLO11n model demonstrates high reliability and stability in practice, offering efficient target recognition and localization capabilities across various classroom behavior detection scenarios.

**Figure 12:**
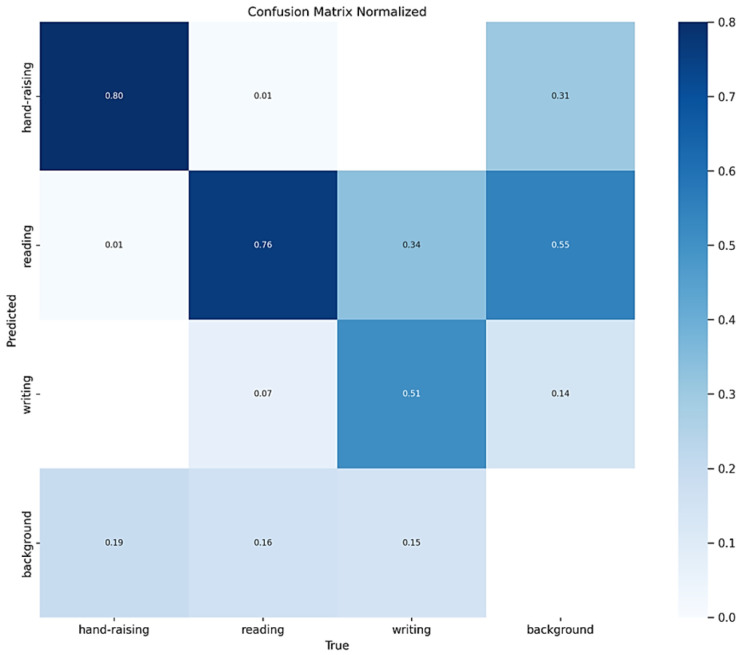
Confusion matrix of the proposed model.

To comprehensively evaluate the practical detection performance of the improved model, we generated a normalized confusion matrix, as shown in Figure 12. This matrix provides a detailed description of the model’s predictive accuracy for three types of student classroom behaviors on the SCB2 dataset, revealing the interrelationships among the predicted categories. In the figure, rows represent the true labels, columns indicate the model’s predicted categories, and the diagonal elements correspond to the proportion of correctly detected instances. An examination of the confusion matrix reveals that the model achieves high accuracy across all behavior categories, with most predictions closely matching the actual labels. This indicates that the proposed improved model can effectively distinguish and recognize different classroom behaviors, demonstrating strong classification capability and robust stability.

It should be noted that the accuracy for the “writing” behavior is slightly lower than that of the other two behaviors, which may be attributed to the smaller number of samples in this category. Nevertheless, the overall results indicate that the model performs well in distinguishing among the three classroom behavior categories. In future work, increasing the number of training samples or incorporating additional discriminative features could further enhance the recognition of the “writing” behavior.

**Figure 13:**
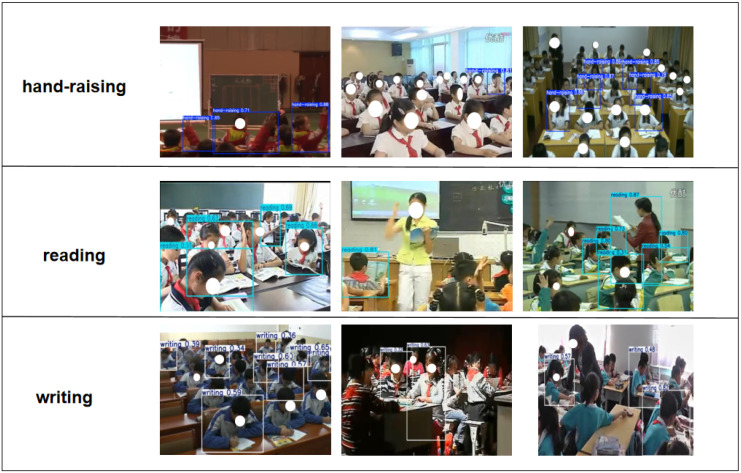
Test results on the SCB2 dataset.

Figure 13 presents the testing results of the PLA-YOLO11n model on the SCB2 dataset. This dataset includes typical challenging scenarios, such as target occlusions and subtle or hard-to-capture actions. As shown in the figure, even under partial occlusion among students or in complex classroom environments, PLA-YOLO11n can stably and accurately identify the main classroom behaviors, demonstrating high robustness and adaptability. In particular, for small or hard-to-capture actions, the model exhibits enhanced detection sensitivity and localization accuracy, effectively addressing the limitations of the original model in recognizing small targets. Overall, PLA-YOLO11n is capable of handling typical detection tasks in real-world classroom scenarios and meets the requirements for high-precision monitoring of student classroom behaviors.

### 4.5. Ablation Experiment

**Figure 14:**
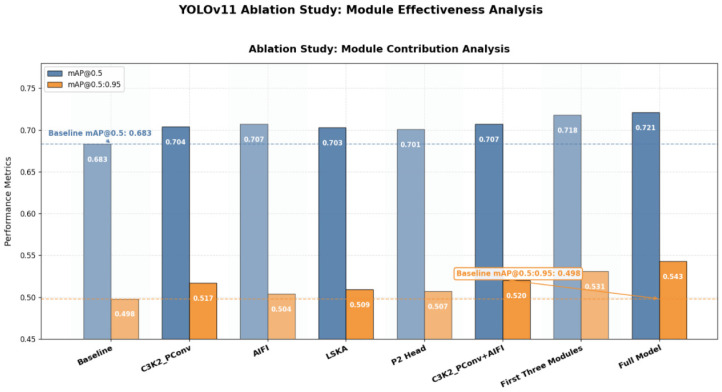
Results of the ablation experiments for PLA-YOLO11n.

To systematically evaluate the contributions of the structural improvement modules to YOLO11’s detection performance, systematic ablation experiments were conducted on the SCB2 dataset, focusing on the individual and combined effects of the four modules: C3K2_PConv, AIFI, LSKA, and the P2 Head. As shown in Figure 14 and Table 3, by progressively integrating the modules and comparing the effects of their removal, the role of each module in enhancing the model’s performance can be clearly validated. The results presented in the figures and tables represent the averages of five independent runs with a fixed random seed of 0, ensuring stability and reliability in performance evaluation.

Table 3 summarizes the contributions of each improvement module to the overall performance as well as to the typical behavior categories (“raising hands”, “reading”, and “writing”). The baseline model achieved AP values of 0.773, 0.717, and 0.553 for the three behavior categories, respectively. After introducing C3K2_PConv, the AP values for the three behaviors increased to 0.798, 0.734, and 0.579, representing the most significant gains, indicating that this module plays a key role in enhancing feature representation and multi-scale adaptability. In contrast, the AIFI and LSKA modules produced the most notable improvements for the “raising hands” category, achieving 0.807 and 0.806, respectively, demonstrating the advantage of attention mechanisms in capturing local action features and distinguishing fine-grained behaviors. Although the isolated introduction of the P2 Head yielded a relatively modest improvement in overall mAP (mAP@0.5 increased to 0.701), it provided consistent gains for the “reading” and “writing” small-scale categories, and additionally improved mAP@0.5:0.95 by 0.012, further demonstrating its unique value in enhancing small-object detection.

Overall, the modules exhibit a clear synergistic effect when combined. When the three modules (C3K2_PConv, AIFI, and LSKA) are integrated jointly, AP, mAP@0.5, and mAP@0.5:0.95 all show significant improvements, with the AP for the “writing” category increasing to 0.608, indicating enhanced discriminative capability in recognizing complex and fine-grained actions. Ultimately, when all four modules are fully integrated to form PLA-YOLO11n, the AP values for the three typical behaviors reach 0.822, 0.751, and 0.591, respectively, while the overall mAP@0.5 increases to 0.721, and mAP@0.5:0.95 rises to 0.543, representing gains of 0.038 and 0.045 over the baseline model.

These results reveal three key findings. First, C3K2_PConv serves as the foundational module for performance improvement, providing the most significant single-module gains, primarily due to its larger receptive field and the adaptability of deformable convolutions across multi-scale scenarios. Second, AIFI and LSKA enhance the model’s feature representation capability through different forms of attention mechanisms, making them particularly effective for distinguishing classroom behaviors with fine-grained differences. Finally, although the P2 Head provides limited improvement on its own, it plays a crucial role in small-object detection and cross-scale information reinforcement, with its value further amplified when integrated synergistically with other modules.

Overall, the synergistic optimization of multiple modules not only improves the overall detection accuracy but also effectively enhances the model’s performance in small-object recognition and occluded scenarios, fully demonstrating the robustness and practical value of PLA-YOLO11n in complex educational settings.

### 4.6. Comparative Experiments

#### 4.6.1. Visualization Results Comparison

**Figure 15:**
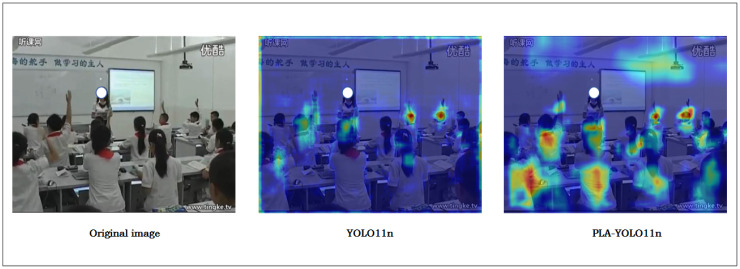
Comparison of heatmaps across different models.

To provide a more intuitive illustration of the PLA-YOLO11n model’s attention to targets, this study employs the Grad-CAM method to generate heatmaps for the original image, the YOLO11n model, and the PLA-YOLO11n model, thereby reflecting their capacity to capture target features. Specifically, classroom images captured from a distance were selected from the SCB2 test set for detection, and the corresponding heatmaps were produced. As shown in Figure 15, the heatmaps adopt a color gradient ranging from blue (indicating low attention) to red (indicating high attention). The more concentrated the red regions are around the target areas, the stronger the model’s focus on the detected objects.

As shown in the heatmaps in Figure 15, the original images depict classroom scenes captured from a distance, containing small-sized targets and some degree of occlusion, such as overlapping students or obstructing objects on desks. The heatmaps of the YOLO11n model display a rather diffuse distribution from blue to green, with sparse red regions appearing only in specific target areas, indicating insufficient attention to small, distant targets and occluded scenes, highlighting limitations in feature extraction. In contrast, the heatmaps of the PLA-YOLO11n model show more concentrated red regions around the target areas, with significantly reduced interference from irrelevant background. This indicates that the PLA-YOLO11n model, through multi-scale feature fusion and contextual modeling, achieves more precise attention to distant small targets and minor occlusions, thereby enhancing feature extraction efficiency and detection robustness. Although distant capture limits the visibility of fine details, the improved model still significantly enhances the localization accuracy of small targets, demonstrating its superior performance in complex classroom scenarios. These comparative heatmaps provide an intuitive reference for researchers, facilitating further optimization and practical application of the proposed method.

#### 4.6.2. Comparative Results Across Different Models

**Figure 16:**
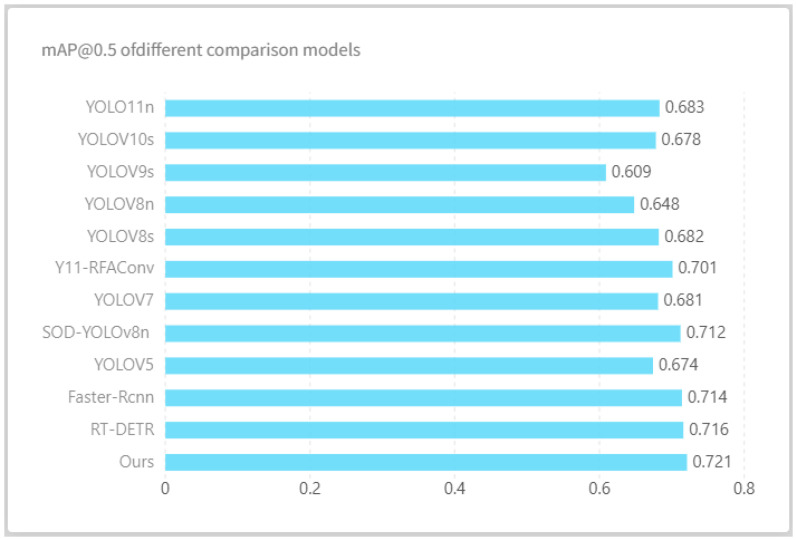
Comparative results across different detection models.

To evaluate the performance of the proposed PLA-YOLO11n model in classroom behavior detection, this study used YOLO11n as the baseline and compared it with several state-of-the-art detection methods. All experiments were conducted on the SCB2 dataset, with models trained under identical training strategies and hardware conditions, a fixed random seed of 0, and five independent runs, with the results averaged to ensure fairness and reliability of comparisons. The experimental results, presented in Figure 16 and Table 4, visually illustrate the detection performance of each model across different behavior categories as well as their overall performance.

Overall, PLA-YOLO11n achieved average precision (AP) values of 0.822, 0.751, and 0.591 for the “raising hands”, “reading”, and “writing” behaviors, respectively, with an overall mAP@0.5 of 0.721, representing a substantial improvement over the baseline YOLO11n value of 0.683. This indicates that the introduced multi-scale feature enhancement, attention modules, and P2 Head effectively strengthen the model’s capability for behavior recognition in complex classroom environments, with particularly notable advantages for the high-interactivity, fine-grained “raising hands” category. Compared with other improved methods such as Y11-RFAConv [36] and SOD-YOLOv8n [37], PLA-YOLO11n demonstrates superior performance across all behavior categories, highlighting its comprehensive advantage in multi-class behavior recognition.

In terms of computational efficiency, the design of PLA-YOLO11n carefully balances multi-scale features with lightweight modules. While the introduction of the P2 head brings a moderate computational overhead, the design of PLA-YOLO11n achieves a careful balance between multi-scale feature representation and lightweight modules. Compared with the substantial improvements in detection accuracy, this additional cost is limited and entirely acceptable. The enhanced model not only preserves a compact parameter size and reasonable computational complexity but also sustains high recognition accuracy, thereby maintaining a relatively high processing speed.

Compared with mainstream detection models, PLA-YOLO11n outperforms commonly used methods such as YOLO11n, YOLOv8, and Faster R-CNN [38] in terms of accuracy, while maintaining a relative advantage in computational efficiency. Even when compared with contemporary Transformer-based methods like RT-DETR [39], PLA-YOLO11n demonstrates a favorable trade-off among overall metrics such as mAP, parameter count, FPS, and FLOPs. This indicates that PLA-YOLO11n can achieve high-precision behavior detection in complex classroom scenarios while maintaining reasonable computational resource usage, highlighting its feasibility and potential for practical application and deployment.

#### 4.6.3. Comparative Results on Different Datasets

To further evaluate the detection performance and generalization capability of the PLA-YOLO11n model across different scenarios, experiments were conducted on two additional datasets: the education-related SCB3-U dataset [35] and the general crowd scenario CrowdHuman dataset [40]. In these experiments, PLA-YOLO11n was trained and tested alongside the baseline YOLO11n model under identical training strategies, with a fixed random seed of 0 and five independent runs, and the results were averaged to ensure stability and comparability.

To assess the model’s performance and generalization capability in educational scenarios, tests were conducted on the SCB3-U dataset. This dataset was specifically constructed for university classroom scenarios, focusing on real student learning behaviors, and contains 671 images with 19,768 behavior annotations, covering six behavior categories: raising hands, reading, writing, using a mobile phone, lowering the head, and resting on the desk. The data were collected from actual classroom recordings and processed using frame interpolation and iterative training to ensure authenticity and applicability. The experimental results are presented in Table 5.

It can be observed that PLA-YOLO11n outperforms the original model in precision, recall, and mAP on this dataset, with mAP@0.5 increasing by approximately 2.6% and mAP@0.5:0.95 improving by about 1.7%, demonstrating a comprehensive enhancement in overall detection performance.

To further evaluate the model’s generalization capability in more complex and diverse scenarios, tests were conducted on the publicly available CrowdHuman dataset, with PLA-YOLO11n compared against the baseline model. This dataset comprises 15,000 training images, 4370 validation images, and 5000 test images, totaling approximately 470,000 pedestrian annotations, with an average of 22.6 pedestrians per image. The images feature varying degrees of occlusion and multi-scale targets. The experimental results are presented in Table 6.

As shown in the table, PLA-YOLO11n also achieves significant advantages on this general crowd dataset, with mAP@0.5 increasing by approximately 2.1% and mAP@0.5:0.95 improving by about 2.0%. Notably, under conditions of high-density crowds and complex occlusions, PLA-YOLO11n maintains high precision and recall, indicating that it is not only suitable for educational scenarios but also capable of delivering stable performance on entirely different types of datasets. This further validates the model’s cross-scenario generalization capability and robustness, providing a reliable foundation for applying PLA-YOLO11n in diverse real-world environments.

## 5. Conclusions and Future Work

We conducted extensive experiments on publicly available datasets, including SCB2, SCB3-U, and CrowdHuman, to comprehensively evaluate the proposed PLA-YOLO11n. The results clearly demonstrate its superior performance in classroom behavior detection. In particular, on the SCB2 dataset, PLA-YOLO11n achieved improvements of 3.8% in mAP@0.5 and 4.5% in mAP@0.5:0.95 compared with the original YOLOv11 model.

The PLA-YOLO11n framework addresses key challenges in classroom behavior detection through systematic architectural optimizations. First, the C3K2_PConv module, introduced into the backbone, leverages partial convolution to enhance feature representation significantly. In the neck, the integration of the LSKA large-kernel attention module effectively improves robustness in detecting behaviors under occlusion. Furthermore, by refining the feature pyramid structure and incorporating an additional P2 high-resolution detection head, the model demonstrates notable improvements in capturing fine-grained and small-scale targets.

To ensure ethical compliance, all experiments were conducted on publicly available datasets (SCB2, SCB3-U, and CrowdHuman), without the use of any newly collected student images. Additionally, all identifiable faces appearing in the datasets were anonymized with mosaic processing to protect individual privacy.

Looking ahead, our future work will focus on further enhancing detection accuracy, streamlining model complexity, and integrating GAN-based image restoration techniques to improve detail recovery from low-quality video frames. These improvements will enable the proposed system to achieve greater precision, efficiency, and stability in diverse classroom scenarios.

## Figures and Tables

**Table 1:** Hardware environment.

Component	Specification
Operating System	Ubuntu 16.04
Processor	104 vCPU Intel Xeon Gold 6230R @ 2.10 GHz
GPU	NVIDIA GeForce RTX 2080 Ti (11 GB)
CUDA	11.8
Python	3.9
PyTorch	2.0.1

**Table 2:** Hyperparameter settings.

Hyperparameter	Value
Epoch	400
Image Size	640 × 640
Batch Size	8
Device	0
Initial Learning Rate	0.01
Optimizer	SGD
Momentum	0.937
Weight Decay	0.0005
Warmup Epochs	3.0
IoU Threshold (Train)	0.20
Confidence Threshold	0.25
Mosaic Augmentation	1.0

**Table 3:** Ablation experiments of PLA-YOLO11n.

C3K2_PConv	AIFI	LSKA	P2	AP			mAP@0.5	mAP@0.5:0.95
				Hand-Raising	Reading	Hand-Raising		
				0.793	0.706	0.793	0.683	0.498
√				0.809	0.724	0.809	0.704	0.518
	√			0.811	0.726	0.811	0.706	0.519
		√		0.807	0.723	0.807	0.703	0.517
			√	0.806	0.721	0.806	0.701	0.516
√	√			0.813	0.728	0.813	0.707	0.52
√	√	√		0.819	0.744	0.819	0.718	0.531
√	√	√	√	0.822	0.751	0.822	0.721	0.543

**Table 4:** Detection results of different models.

Model	AP			mAP@0.5	FPS	Params (M)	FLOPs (G)
	Hand-Raising	Reading	Writing				
YOLO11n	0.793	0.706	0.551	0.683	84	84	2.58
YOLOV10s	0.776	0.692	0.564	0.678	225	225	8.07
YOLOV9s	0.741	0.614	0.472	0.609	251	251	6.32
YOLOV8n	0.766	0.672	0.507	0.648	283	283	3.01
YOLOV8s	0.792	0.691	0.564	0.682	243	243	9.84
Y11-RFAConv	0.805	0.731	0.566	0.701	106	106	2.6
YOLOV7	0.788	0.696	0.559	0.681	94.2	94.2	36.5
SOD-YOLOv8n	0.817	0.741	0.578	0.712	139	139	3.24
YOLOV5	0.775	0.705	0.542	0.674	175	175	7.09
Faster-Rcnn	0.817	0.74	0.586	0.714	27.1	27.1	137.1
RT-DETR	0.815	0.746	0.587	0.716	67.0	67.0	198.0
Ours	0.822	0.751	0.591	0.721	184	184	3.08

**Table 5:** Comparison of detection results on the SCB3-U dataset.

Model	P	R	mAP@0.5	mAP@0.5:0.95
YOLO11n	0.86	0.789	0.738	0.496
PLA-YOLO11n	0.871	0.824	0.764	0.513

**Table 6:** Comparison of detection results on the CrowdHuman dataset.

Model	P	R	mAP@0.5	mAP@0.5:0.95
YOLO11n	0.812	0.767	0.721	0.492
PLA-YOLO11n	0.829	0.783	0.742	0.512

## Data Availability

Data available in a publicly accessible repository: The original data presented in the study are openly available in GitHub (SCB-dataset repository) at https://github.com/Whiffe/SCB-dataset, accessed on 26 August 2025, and are additionally documented in the research publication available through arXiv at https://doi.org/10.48550/arXiv.2304.02488, accessed on 26 August 2025.
